# Biological Networks Entropies: Examples in Neural Memory Networks, Genetic Regulation Networks and Social Epidemic Networks

**DOI:** 10.3390/e20010036

**Published:** 2018-01-13

**Authors:** Jacques Demongeot, Mariem Jelassi, Hana Hazgui, Slimane Ben Miled, Narjes Bellamine Ben Saoud, Carla Taramasco

**Affiliations:** 1 Team AGIM (Autonomy, Gerontechnology, Imaging, Modelling & Tools for e-Gnosis Medical), Laboratory AGEIS, University Grenoble Alpes, Faculty of Medicine, La Tronche 38700, France; 2 Escuela de Ingeniería Civil en Informática, Universidad de Valparaíso, General Cruz 222, Valparaíso 2340000, Chile; 3 Laboratory of Bioinformatics, Biomathematics and Biostatistics (BIMS), Institut Pasteur de Tunis, Tunis 1002, Tunisia; 4 National School for Computer Studies, RIADI Laboratory, University of Manouba, Manouba 2010, Tunisia

**Keywords:** biological networks, dynamic entropy, isochronal entropy, attractor entropy, entropy centrality, robustness

## Abstract

Networks used in biological applications at different scales (molecule, cell and population) are of different types: neuronal, genetic, and social, but they share the same dynamical concepts, in their continuous differential versions (e.g., non-linear Wilson-Cowan system) as well as in their discrete Boolean versions (e.g., non-linear Hopfield system); in both cases, the notion of interaction graph *G(J)* associated to its Jacobian matrix *J*, and also the concepts of frustrated nodes, positive or negative circuits of *G(J)*, kinetic energy, entropy, attractors, structural stability, etc., are relevant and useful for studying the dynamics and the robustness of these systems. We will give some general results available for both continuous and discrete biological networks, and then study some specific applications of three new notions of entropy: (i) attractor entropy, (ii) isochronal entropy and (iii) entropy centrality; in three domains: a neural network involved in the memory evocation, a genetic network responsible of the iron control and a social network accounting for the obesity spread in high school environment.

## 1. Introduction

Biological networks are currently widely used to represent the mechanisms of reciprocal interaction between different biological entities and at different scales [1,2,3]. For example, if the entities are molecular, the networks can concern the genes and the control of their expression, or the enzymes and the regulation of the metabolism in living systems that possess them. At the other end of the life spectrum, at the population level, biological networks are used to model interactions between individuals, for example during the spread of contagious diseases, whether social or infectious [4,5]. Between these two molecular and population levels, the cellular level is also concerned by biological networks: for example, in the same neural tissue, cells such as neurons work together to give rise to emergent properties, such as those of the central nervous system related to memory [6] or motion control [7].

Among the tools used for quantifying the complexity of a biological network, the network entropies are ones of the most easy to calculate. They can be related to the network dynamics, i.e., calculated from the Kolmogorov-Sinaï formula when the dynamics is Markovian, or they can represent the richness of the network attractors or confinors [8], based on the relative weight of their attraction or confinement basins in the network state space. A final way to calculate the entropy of a random network is to use the invariant measure of its dynamics, giving birth to probability measures related to the characteristics of its nodes like centrality, connectivity, strong connected components size, etc. The literature on network entropies is abundant and from reference to recent papers it is possible to find definitions and calculation algorithms in [9,10,11,12,13,14,15,16,17,18,19,20,21,22].

Dynamical systems, discrete or continuous, share common mathematical notions like initial conditions and attraction basin, final conditions and attractor, Jacobian graph, stability and robustness. Moreover, in an energetic view, there are in both cases, two extreme systems, i.e., on one hand, conservative systems with an energy constant along trajectories (e.g., Hamiltonian systems) and, on the other hand, dissipative systems with an energy decreasing along trajectories (e.g., potential systems). For both Hamiltonian and potential systems, it is possible to define at each point x of their state space E, a Jacobian matrix *J(x)* made of the partial derivatives of the flow of the network dynamics, whose associated graph having *J(x)* as incidence matrix, is called the interaction graph *G(x)*. The description of the dynamics in terms of energy conservation or dissipation is identical whether the system is time discrete or continuous.

Common definitions of the concepts of attraction basin and attractor will be recalled in Section 2 Material and Methods, in which we give also some examples of Hamiltonian and potential systems by making explicit the energy functions underlying their dynamics, allowing for interpreting these functions in the precise context of different applications. For example, the energy functions can be related to the notions of frustration in statistical mechanics [23] or of kinetic energy, which can be calculated in discrete systems by using an analogue of the continuous velocity [24]. In Section 3, we present the Results through applications in three domains, neural (memory evocation based on the synchronization of neural networks), genetic (regulation of the iron metabolism) and social (dynamics of the obesity spread in a college environment). We define the thermodynamical notions of attractor entropy, dynamic (or evolutionary) entropy and entropy centrality for interpreting the results, and we relate the dynamic entropy to the robustness (or structural stability) of the dynamical systems presented. After, in Section 4, the Discussion presents the interest of a thermodynamic approach in social networks in order to simulate and evaluate the efficiency of public health policies, and eventually, the Section 5 gives the Conclusion and Perspectives of the paper.

## 2. Material and Methods

### 2.1. The Dynamical Tools

Several definitions of attractor and stability have been proposed since 50 years. Here we use that of Cosnard and Demongeot [25,26], which covers both continuous and discrete cases. More specific approaches of the notion of attractors especially for network dynamics can be found in [3,27,28,29].

#### 2.1.1. Definition of the Notion of Attractor

Let consider a trajectory ${\left\{f(a,t)\right\}}_{t\in T}$ starting at the point a of the state space $E\subset R^{n}$ provided with a dynamic flow *f* and a distance *d*. L(a), called the limit set of the trajectory starting in *a*, is the set of accumulation points of the trajectory ${\left\{f(a,t)\right\}}_{t\in T}$ as time *t* goes to infinity in the time set *T* (either discrete or continuous) such as:

$$L\left(a\right)=\left\{y\in E;\forall \varepsilon >0,\forall t\in T,\exists s(\varepsilon ,t)>t/d(f(a,s(\varepsilon ,t)),y)<\varepsilon \right\}$$

where s(ε,t) is one time greater than t for which the trajectory passes into a ball of radius ε centred in *y*.

If the initial state a lies in a set *A*, L(A) is the union of all limit sets L(a), for *a* belonging to *A*:

$$L\left(A\right)=\cup_{a\in A}L\left(a\right)$$

**Figure 1:**
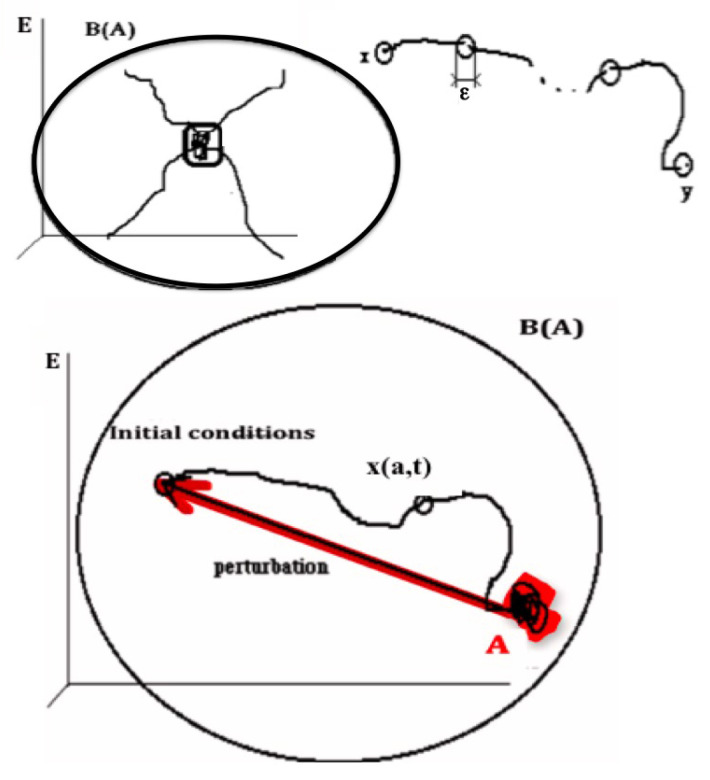
Top left : Attractor A is invariant for the composed operator LoB. Top right: Shadow trajectory between x and y. Bottom: State of the attractor A returning to A after a perturbation in the attraction basin B(A).

Conversely, B(A) is the set of all initial conditions *a*, whose limit set L(a) is included in *A* (Figure 1). $B\left(A\right)=\left\{y\in E;L(y)\subset A\right\}$ is called the attraction basin of *A*.

(i) A=LoB(A), where the composed operator LoB is obtained by applying the basin operator *B* and then the limit operator *L* to the set *A*
(ii) There is no set *A*’ containing strictly *A*, shadow-connected to *A* and verifying (i)
(iii) There is no set *A*” strictly contained in *A* verifying (i) and (ii)

The definition of the “shadow-connectivity” connecting in *E* the subsets $F_{1}$ and $F_{2}$, lies on the fact that there exists a “shadow-trajectory” between $F_{1}$ and $F_{2}$ (Figure 1 Top right, where $F_{1}=\left\{x\right\}$ and $F_{2}=\left\{y\right\}$). The notion of “shadow-trajectory” has been defined first in [30]:

*x* in $F_{1}$ and *y* in $F_{2}$ belong to the same shadow-trajectory between $F_{1}$ and $F_{2}$ if, for any ε>0, there is an integer n(ε) and n(ε) times t(1,ε),…,t(n(ε),ε) and states y(1,ε),…,y(n(ε),ε), such as all Hausdorff distances between the successive parts of the shadow-trajectory are less than ε:

d(f(x,t(1,ε)),y(1,ε))≤ε,…,d(f(y(k−1,ε),t(k−1,ε)),y(k,ε))≤ε,…,d(f(y(n(ε),ε),t(n(ε),ε)),y)≤ε

The above definition of attractor is available for all known dynamical systems, in agreement with the common sense and is meaningful for both discrete or continuous time sets T, if the dynamical system is autonomous with respect to time, contrary to other definitions proposed since 1970 such as in [31,32,33,34], all less general.

#### 2.1.2. Potential Dynamics

A dynamical system is potential (or gradient) if its velocity is equal to the gradient of a scalar potential *P* defined on the state space *E*: ∂x(t)/∂t=−gradP=−∂P/∂x where gradient vector equals $\partial P/\partial x=(\partial P/\partial x_{1},\ldots ,\partial P/\partial x_{n})$ and state at time *t* is $f\left(x\left(0\right),t\right)=x\left(t\right)=\left(x_{1}\left(t\right),\ldots ,x_{n}\left(t\right)\right)$ [35]. The system is dissipative, because *P* decreases along trajectories until its attractors located on its minima.

#### 2.1.3. Hamiltonian Dynamics

A dynamical system is Hamiltonian if its velocity is tangent to the projection on *E* of the contour lines of the surface representative of an energy function *H* on *E*: ∂x(t)/∂t=tangH(x(t)), where tangH(x(t)) is the vector tangent at x(t) to the projection on *E* of the equi-altitude set of altitude H(x(t)). If the dimension of the system is two, then tangH is equal to $\left(\partial H/\partial x_{2},-\partial H/\partial x_{1}\right)$ [36]. The system is said conservative, because the energy function *H* is constant along trajectories. An example of conservative system will be given in Section 4.2 (Equation (16)).

#### 2.1.4. Potential-Hamiltonian Dynamics

A dynamical system is potential-Hamiltonian if its velocity can be decomposed into two parts, one potential and one Hamiltonian: ∂x(t)/∂t=−gradP+tangH [37]. If the set of minima of *P* is a contour line of the surface *H* on *E*, then its strong shadow-connected components (i.e., the sets in which any pair of states is shadow-connected in both senses) are the attractors of the system.

### 2.2. Examples of Dissipative Energy

In a gradient system, the potential energy is dissipative, because it decreases along the trajectories and, more, its gradient is exactly equal to the system velocity. The existence of a morphogenetic dissipative energy has been speculated by the Waddington’s school of embryology [38] as an ontology rule governing the development of living systems and we give in Section 2.2.2 an example of such a dissipative energy.

#### 2.2.1. Discretization of the Continuous Potential System with Block-Parallel Updating

Let us consider a discrete Hopfield dynamics where $x_{i}\left(t\right)=1$, if the gene *i* is expressing its protein, else $x_{i}\left(t\right)=0$. The probability that $x_{i}\left(t\right)=1$, knowing the states in an interaction neighbourhood $V_{i}$ of *i* is given by:

(1) $$P\left(\left\{x_{i}\left(t\right)=1\right\}|\left\{x_{j}\left(t-1\right),j\in V_{i}\right\}\right)=\exp\left(\left(\sum_{j\in V_{i}}w_{ij}x_{i}\left(t-1\right)-\theta \right)/T\right)$$

where $w_{ij}$ is a parameter which represents the influence (inhibitory, if $w_{ij}<0$; activatory, if $w_{ij}>0$ and null, if $w_{ij}=0$) the gene *j* from the interaction neighbourhood $V_{i}$ is exerting on the gene *i*, and θ is a threshold corresponding to the external field in statistical mechanics and here to a minimal value of activation for the interaction potential $\sum_{j\in V_{i}}w_{ij}x_{i}$. *T* is a temperature allowing for introducing a certain degree of indeterminism in the rule of the change of state. In particular, if T=0, the rule is practically deterministic and, if *H* denotes the Heaviside function, we have:

(2) $$\begin{array}{c} x_{i}\left(t\right)=1,\;\mathrm{if}\sum_{j\in V_{i}}w_{ij}x_{i}\left(t-1\right)>\theta \\ x_{i}\left(t\right)=0,\;\mathrm{if}\sum_{j\in V_{i}}w_{ij}x_{i}\left(t-1\right)<\theta \end{array}\}x_{i}\left(t\right)=H\left(\sum_{j\in V_{i}}w_{ij}x_{i}\left(t-1\right)-\theta \right)$$

$$P\left(\left\{x_{i}\left(t\right)=1\right\}|\left\{x_{j}\left(t-1\right),j\in V_{i}\right\}\right)=1/2,\mathrm{if}\sum_{j\in V_{i}}w_{ij}x_{i}\left(t-1\right)=\theta$$

Once this rule defined, three graphs and their associated incidence matrices are important for making more precise the network dynamics. These incidence matrices are:

- interaction matrix *W*: $w_{ij}$ represents the action of the gene *j* on gene *i*. *W* plays the role of a discrete Jacobian matrix. We can consider the associated signed matrix *S*, with $s_{ij}=1$, if $w_{ij}>0$, $s_{ij}=-1$, if $w_{ij}<0$ and $s_{ij}=0$, if $w_{ij}=0$,
- updating matrix *U*: $u_{ij}=1$, if *j* is updated before or with *i*, else $u_{ij}=0$,
- trajectory matrix *F*: $f_{bc}=1$, where *b* and *c* are two states of *E*, if and only if b=f(c,1), else $f_{bc}=0$.

These matrices can be constant or can depend on time *t*:

- in the case of W, the most frequent dependence is called the Hebbian dynamics: if the vectors ${\left\{x_{i}\left(s\right)\right\}}_{s<t}$ and ${\left\{x_{j}\left(s\right)\right\}}_{s<t}$ have a correlation coefficient $\rho_{ij}\left(t\right)$, then $w_{ij}\left(t+1\right)=w_{ij}\left(t\right)+h\rho_{ij}\left(t\right)$, with h>0, corresponding to a reinforcement of the absolute value of the interactions $w_{ij}$ having succeeded in inhibiting or activating their target gene *i*: in case where, for s<t, $x_{j}\left(s\right)$ remained equal to one, that leads to increase the $x_{i}\left(s\right)$’*s*, if the $w_{ij}\left(s\right)$’*s* were positive, and conversely to decrease the $x_{i}\left(s\right)$’*s* , if the $w_{ij}\left(s\right)$’*s* were negative,
- in the case of U, we can have an autonomous (in time) clock based on the behaviour of *r* chromatin clock genes having indices 1,…,r, with three possible behaviours:
  1. if $y\left(t\right)=\prod_{i=1,\ldots ,r}x_{i}\left(t\right)=1$, then the rule (1) is available
  2. if y(t)=0 and $\sum_{s=t,\ldots ,t-c}y\left(s\right)>0,x\left(t+1\right)=x\left(s^{*}\right)$, where $s^{*}$ is the last time between t−c and *t*, where $y(s^{*})=1$
  3. if y(t)=0 and $\sum_{s=t,\ldots ,t-c}y\left(s\right)=0$, then x(t+1)=0 (by exhaustion of the pool of expressed genes).

This dynamical system ruling the evolution of U remains autonomous, but the updating is state dependent. If all $u_{ij}$ equal one, the updating is called parallel; if there is a sequence $i_{1},\ldots ,i_{n}$ such as $u_{kk+1}=1$ for any *k* such as 1≤k≤n−1, the other $u_{ij}$ being equal to zero, then the updating is called sequential. There exist cases other than these extreme cases called block-sequential (the updating is parallel in a block and the blocks are updated sequentially). A case of updating which is more realistic in genetics is called block parallel: there are blocks made of genes sequentially updated, and these blocks are updated in parallel. Interactions between these blocks can exist (i.e., there are $w_{ij}\neq 0$, with *i* and *j* belonging to two different blocks), and because the block sizes are different, the time at which the stationary state is reached in a block is not necessarily synchronized with the corresponding times in other blocks: the synchronization depends on the intra-block as well as on the inter-block interactions. All the genes of a block can reach their stationary state long before the genes of another block. This behaviour explains in particular that the state of the genes of the block corresponding for example to the chromatin clock is highly depending on the state of the genes in other blocks connected to them (and for example acting as transcription factors of the clock genes), and reciprocally.

**Figure 2:**
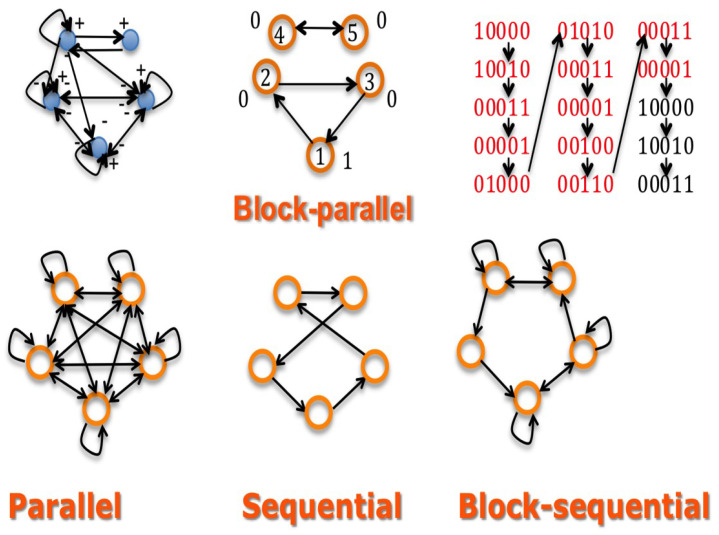
Top left: interaction graph of a network made of a 3-switch (system made of three genes fully inhibited except the auto-activations) representing morphogens linked to a regulon representing chromatin clock genes. Top middle: the updating graph corresponding to a block-parallel dynamics ruling the network. Top right: a part of the trajectory graph exhibiting a limit-cycle of period 12 having internally a cycle of period four for the chromatin clock genes. Bottom: updating graphs corresponding successively (from the left to the right) to the parallel, sequential and block-sequential dynamics.

An example of such a block-parallel dynamics is given by a short block made of the nodes 4 and 5 on Figure 2 Top middle of chromatin clock genes reaching rapidly its limit-cycle and entraining other blocks of metabolic (nuclear or mitochondrial) genes. The second block of genes is made of the morphogens responsible of the apex growth of a plant and of the auxin gene inhibiting the morphogens (node 1) [39,40]. When the first block reaches its limit-cycle (possibly entrained by external “Zeitgebers” related to an exogenous seasonal rhythm), it blocks the dynamics of the second block, liberating the first and after, the second axillary bud morphogens (nodes 2 and 3) only when the apex morphogens passed a sufficient time in state 1, corresponding to a sufficient growth of apex for limiting the diffusion of auxin, then cancelling its inhibitory power. We can easily iterate this dynamical behaviour for other successive blocks related to the inferior buds.

More precisely, we can implement the dynamics of the morphogenesis network in the following way; let us suppose that:

(i) there are two chromatin clock genes involved in a regulon, i.e., the minimal network having only one negative circuit and one positive circuit reduced to an autocatalytic loop
(ii) there are three morphogens corresponding to the apex and to axillary buds involved as nodes of a 3-switch network fully connected, with all interactions negative except three autocatalytic loops at each vertex
(iii) there are three inhibitory interactions from the auto-catalysed node of the regulon on the nodes of the 3-switch, as indicated on Figure 2 Top left.

The Figure 2 Top shows the three graphs defining the morphogenesis network dynamics, i.e., the interaction (left), updating (middle) and trajectory (right) ones. The dynamics is supposed ruled by a deterministic Hopfield network, with temperature and threshold equal to zero and interaction weights $w_{ij}$ equal to one (if positive), −1 (if negative) or zero. An attractor limit-cycle of this dynamics is shown on Figure 2 Top right, showing two intricate rhythms, one of period four corresponding to the chromatin clock dynamics, embedded in a rhythm of period 12 corresponding to the successive expression of the apex and axillary buds morphogens. The Figure 2 Bottom shows other updating graphs, less realistic than the block-parallel for representing the action of the chromatin clock regulon.

#### 2.2.2. Discrete Lyapunov Function (Neural Network)

**Figure 3:**
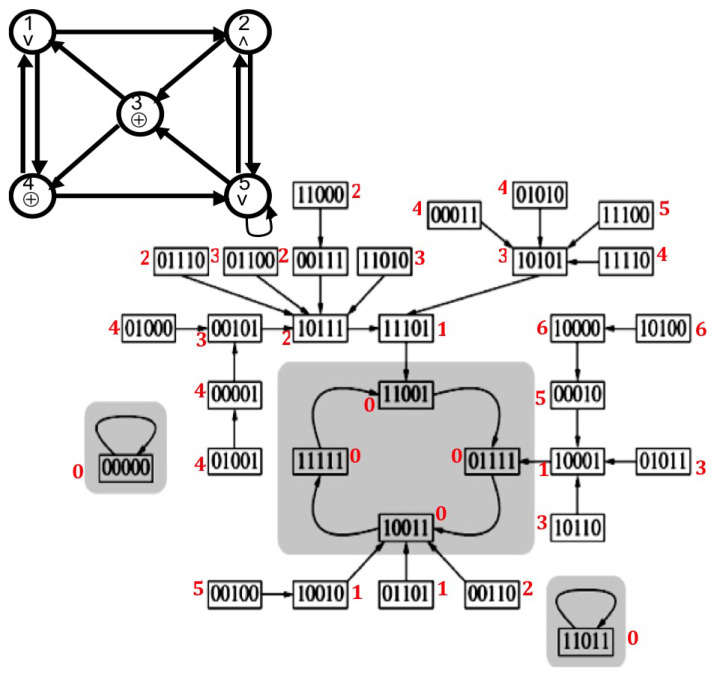
Top: Logic neural network with local transition rules ⊕, ∨ and ∧. Bottom: Discrete trajectory graph in the state space $E={\left\{0.1\right\}}^{5}$ with indication of the values of 64L, where *L* is the Lyapunov function (in red).

A logic neural network has local transition rules of type ⊕, ∨ and ∧ (Figure 3). Applying these local rules ∧ (“and”), ∨ (inclusive “or”) et ⊕ (exclusive “or”), we can define on each configuration x(t) of the logic neural network of the Figure 3, a Lyapunov function *L* from the global frustration of order four $D_{4}$, decreasing along trajectories and vanishing on the attractors of the network dynamics:

$$L\left(x\left(t\right)\right)=\left[D_{4}\left(x\left(t+1\right)\right)+D_{4}(x\left(t\right)\right]/64$$

with $D_{4}\left(x\left(t\right)\right)=\sum_{i=1,\ldots ,n}D_{4,i}\left(x\left(t\right)\right)$, where $D_{4,i}$ is the local frustration of order four, also equal to the discrete kinetic energy $E_{4,i}\left(x\left(t\right)\right)$ between $x_{i}\left(t\right)$ et $x_{i}\left(t+4\right)$ multiplied by 32:

$$D_{4,i}\left(x\left(t\right)\right)/32={\left(x_{i}\left(t+4\right)-x_{i}\left(t\right)\right)}^{2}/2{\left(t+4-t\right)}^{2}=E_{4,i}\left(x\left(t\right)\right)$$

In this example of a discrete neural network ruled by logic Boolean functions applied for each node to its entries (proposed in [41,42] as a genetic network), the dynamical behaviour is guided by an energy function deriving from the discrete kinetic energy, built with the discrete equivalent of the velocity [24]. We can indeed write the Lyapunov function of the trajectory L(x(t)) as the mean global discrete kinetic energy calculated between times *t* and t+5:

$$L\left(x\left(t\right)\right)=\left[D_{4}\left(x\left(t+1\right)\right)+D_{4}(x\left(t\right)\right]/64=\left[E_{4}\left(x\left(t+1\right)\right)+E_{4}\left(x\left(t\right)\right)\right]/2$$

## 3. Results

### 3.1. Attractor Entropy and Neural Networks

The attractor entropy $E_{attractor}$ measures the heterogeneity of the attractor landscape on the state space *E* of a dynamical system. It can be evaluated by the quantity:

(3) $$E_{attractor}=-\sum_{k=1,\ldots ,m}RABS\left(A_{k}\right)Log\left[RABS(A_{k})\right]$$

where $RABS(A_{k})$ is equal to the attraction basin size of the *k*th attractor $A_{k}$ among the *m* attractors of the network dynamics, divided by $2^{n}$, *n* being the number of genes of the network. We will give an example of use of this notion in the case of a continuous neural network, the Wilson-Cowan oscillator, which describes the biophysical interactions between two populations of neurons:

(4) $$dx/dt=-x/\tau_{x}+tanh\left(\lambda x-\theta \right)\text{–}tanh\left(\lambda y-\theta \right),dy/dt=-y/\tau_{y}+tanh\left(\lambda x-\theta \right)+tanh\left(\lambda y-\theta \right)$$

**Figure 4:**
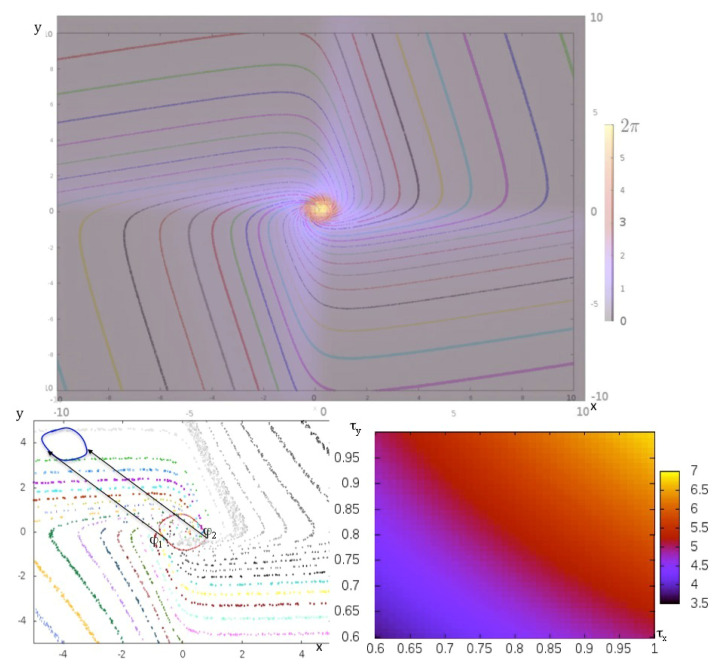
Top: asymptotic phase shift between two close isochrons in false colors from 0 to 2π, when an instantaneous perturbation is made on the Wilson-Cowan oscillator. Bottom left: perturbations of same intensity made at two different phases $\varphi_{1}$ and $\varphi_{2}$ on the limit-cycle of the Wilson-Cowan oscillator. Bottom right: value of the period of the limit-cycle in false colors depending on the values of the parameters $\tau_{x}$ and $\tau_{y}$ from 3.5 to 7.

The variables x and y represent the global electrical activity of the two populations, respectively excitatory and inhibitory, whose interaction is described through a sigmoid function parametrized by λ, which controls its stiffness, accounting for the synaptic response to an input signal, which is non-linear when λ is large (>>1), depending on the value of the input, greater or less than a potential barrier (here θ/λ), and is an affine function of the input signal when λ and θ are small (<<1). The parameters $\tau_{x}$ and $\tau_{y}$ refer to the membrane potential characteristics of the neuronal populations having respective activity x and y. If θ=0 and $\tau_{x}=\tau_{y}=\tau$, the Wilson-Cowan oscillator presents a Hopf bifurcation when λτ crosses the value one [43], moving from a dynamics involving only an attracting fixed point to a dynamics comprising a repulsor at the origin of *E*, surrounded by an attractor limit-cycle C. If *T* denotes the period of the limit-cycle C, let us consider the point h=f(O,h) of C reached after a time (or phase) *h* on the trajectory *f* starting on C at a reference point called the origin O=f(O,0). In Figure 4 Top, T=2π and this trajectory on C defines a one-to-one map *F* between the interval [0,T] and C. We introduce now the notion of isochron $I_{h}$ of phase *h* in the attraction basin B(C) as the set of points *x* of B(C) such as d(f(x,t),f(h,t)) tends to zero, when t tends to infinity. $I_{h}$ extends the map *F* to all the points of *B*(*C*). If we consider as new state space $I_{h}$, a new time space $T_{h}=\left\{t\in T;t=h+kT,k\in N\right\}$ and the restriction of the flow *f* on $I_{h}$, such as: $\forall x\in I_{h},\;f\left(x,t\right)\in I_{h},\;h=lim_{t\in T_{h};t\to \infty }f\left(x,t\right)$, we have: $B\left(C\right)=\cup_{h\in C}I_{h}$.

Then, *h* can be considered as an attractor for *f*, whose basin is $I_{h}$. Let us decompose now the interval [0,T] in n equidistant sub-intervals ${\left\{[kT/n,(k+1)T/n]\right\}}_{k=0,\ldots ,n-1}$. If we consider now the time space [kT/n,(k+1)T/n], the subset of C denoted $A_{k}=F\left(\left[kT/n,\left(k+1\right)T/n\right]\right)$ is an attractor with an attraction basin equal to $B_{k}=\cup_{h\in ([kT/n,(k+1)T/n])}I_{h}$, for the flow restrained to $B_{k}$, and we have: $B\left(C\right)=\cup_{k=0,\ldots ,n-1}B_{k}$. Eventually, we can define the entropy related to these attractors in E, called isochronal entropy of degree *n*, which is just the attractor entropy calculated for the dynamics having as flow $f_{h}$:

(5) $$E_{attractor}^{n}=-\sum_{k=1,\ldots ,n}RABS\left(A_{k}\right)Log\left[RABS\left(A_{k}\right)\right]$$

If all the isochron basins have same size, then $E_{attractor}^{n}=Logn$. If not, the quantity $E_{attractor}^{n}$ reflects a spatial heterogeneity of the dynamics: certain $B_{k}$ can be wide (where the flow *f* is rapid inside) and other thin (where the flow *f* is slow inside). In the example of the Wilson-Cowan oscillator, when $\tau_{x}=\tau_{y}=1$, θ=0 and λ is growing from zero to infinity, the isochronal entropy is diminishing from Logn to Log4.

It is possible to change the value of the period *T* of the Wilson-Cowan oscillator by tuning the parameters $\tau_{x}$ and $\tau_{y}$ (Figure 4 Bottom right). Consider now values of the parameters, $\tau_{x}=\tau_{y}=\tau =1$ and λ=1.1, close to the bifurcation in the parameter space (λτ) and superimpose the isochron landscape on a map in false color giving the asymptotic phase shift $\left|\right|h^{\prime }-h\left|\right|$ between a trajectory starting at a point x on the isochron $I_{h}$ and a trajectory starting, after an instantaneous perturbation e, at the point x+e belonging to the isochron $I_{h^{\prime }}$ (Figure 4 Bottom left). The maximum phase shift of the Wilson-Cowan oscillator is inversely proportional to the intensity of this instantaneous perturbation, which means that a population of oscillators can then be synchronized by a sufficiently intense perturbation. Indeed, isochrons are spirals, which diverge from one to another by moving away from the attractor limit-cycle. The phenomenon of memory evocation after a sensory stimulation relies on the synchronizability of a population of identical (but dephased) oscillators whose successive states of their limit cycle store an information [44,45]. This evocation is the more effective since all oscillators have the same phase after stimulation, which is the case if the corresponding perturbation done at different phases $\varphi_{1}$ and $\varphi_{2}$ on the limit-cycle leads the oscillator between two successive isochrons (Figure 4 Bottom left). By using either the discrete version of the Wilson-Cowan system, namely the Kauffman-Thomas cellular automaton (the deterministic version of the Hopfield model), or its continuous version (Figure 4), it is easy to see that the potential part of the system is responsible for the spacing between isochrons away from the limit-cycle, causing the decrease of the isochronal entropy, and that the Hamiltonian part is responsible for their spiralization (Figure 4 Top).

By playing with the potential-Hamiltonian distribution [46], it is therefore possible, if the system is instantly perturbed, to increase the efficiency of the post-perturbation synchronization (due to the potential return to the limit-cycle) or to allow for desynchronizing (due to the Hamiltonian spiralization), which is necessary to avoid the perseveration in a synchronized state (observed in many neuro-degenerative pathologies).

### 3.2. Dynamic Entropy and Genetic Networks

We define first the functions energy *U*, frustration *F* and dynamic entropy *E* of a genetic network *N* with *n* genes in interaction [47,48,49,50,51,52,53,54,55,56,57,58,59].

(6) $$\forall x\in \Omega ,U\left(x\right)=\sum_{i,j\in \left\{1,n\right\}}\alpha_{ij}x_{i}x_{j}=Q_{+}\left(N\right)-F\left(x\right),$$

where *x* is a configuration of gene expression ($x_{i}=1$, if the gene *i* is expressed and $x_{i}=0$, if not), Ω denotes the set of all configurations of gene expression (i.e., the hypercube ${\left\{0,1\right\}}^{n}$) and $\alpha_{ij}$ is the sign of the interaction weight quantifying the influence the gene *j* exerts on the gene *i*: $\alpha_{ij}=+1$ (resp. −1), if *j* is an activator (resp. inhibitor) of the expression of *i*, and $\alpha_{ij}=0$, if there is no influence of *j* on *i*. $Q_{+}\left(N\right)$ is the number of positive edges of the interaction graph *G* of the network *N*, whose incidence matrix is given by $A={\left(\alpha_{ij}\right)}_{i,j\in \left\{1,n\right\}}$. F(x) denotes the global frustration of *x*, i.e., the number of pairs of genes (i,j) for which the values of $x_{i}$ and $x_{j}$ are contradictory with $\alpha_{ij}$, the sign of the interaction of *j* on *i*:

$$F\left(x\right)=\sum_{i,j\in \left\{1,n\right\}}F_{ij}\left(x\right),$$

where $F_{ij}$ is the local frustration of the pair (i,j) equal to:

$$\left\{\begin{array}{c} 1,\;\mathrm{if}\;\alpha_{ij}=1,\mathrm{with}\;x_{j}=1-x_{i},\;\mathrm{or}\;\alpha_{ij}=-1,\;\mathrm{with}\;x_{j}=x_{i}\\ 0,\;\mathrm{elsewhere} \end{array}\right.$$

The dynamics of the network can be defined by a deterministic transition operator like the following threshold Boolean automaton with a majority rule, we will call in the following deterministic Hopfield transition:

(7) $$x_{i}\left(t\right)=H\left(\sum_{j\in N_{i}}w_{ij}x_{j}\left(t\right)-\theta_{i}\right),$$

where *H* is the Heaviside step function defined by: H(y)=1, if y>0; H(y)=0, if y≤0, $w_{ij}$ is the interaction weight of *j* on *i*, $\theta_{i}$ an activation threshold (whose exponential is called tolerance *h*: θ=logh) and $N_{i}$ is the set of genes *j* such as $w_{ij}\neq 0$. The Table 1 shows the parallel simulation of such a Boolean automaton, with $|w_{ij}|=1$ and $\theta_{i}=0$. This deterministic dynamics is just a particular case of the random dynamics defined by the transition analogue to the Equation (7):

(8) $$m_{ij}=P\left(\left\{x_{i}\left(t\right)=1\right\}|\left\{x(t-1)=y\right\}\right)=exp\left[\sum_{j\in \{1,n\}}w_{ij}y_{j}/T\right]=P_{i,1}^{\left\{i/y_{i}=1\right\}},$$

when threshold and temperature equal to zero.

$m_{ij}$ is the probability of expression of the gene *i* at time *t*, knowing the configuration at time t−1 is equal to y and from the ${\left\{m_{ij}\right\}}_{i,j\in \{1,n\}}$ we can easily calculate the Markov transition matrix of the dynamics (8) supposed to be sequential [48,60], i.e., with a sequential updating of the genes in a predefined order.

Let $M=(M_{xy})$ denote the Markov matrix giving the transition probabilities, defined by the rule (8) and an updating mode, between the configurations *x* and *y* of Ω, and $\mu =\left(\mu_{x}\right)=(\mu {\left(\left\{x\right\}\right)}_{x\in \Omega }$ be its stationary distribution on Ω. The dynamic entropy E can be calculated as:

$$E=-\sum_{x,y\in \Omega }\mu_{x}M_{xy}logM_{xy}.$$

In the sequential updating mode, where the updating order of the nodes is the integer ranking 1,…,n, we have, by denoting $I=\left\{1,\ldots ,i-1\right\},N\backslash I=\left\{i,\ldots ,n\right\}$ and identifying *x* with the set of the indices *i* such that $x_{i}=1$:

$$M_{xy}=\prod_{i=1,\ldots ,n}\left[P_{i,1}^{\left[x\cap (N\backslash I)\right]\cup \left[y\cap I\right]}I_{\left\{i\in y\right\}}+P_{i,0}^{\left[x\cap (N\backslash I)\right]\cup \left[y\cap I\right]}I_{\left\{i\notin y\right\}}\right]$$

and μ is classically the Gibbs measure [48,60] defined by:

$$\forall x\in \Omega ,\mu_{x}=exp\left(\left(\sum_{i\in x,j\in N_{i}}w_{ij}x_{i}x_{j}-\theta_{i}\right)/T\right)/Z,$$

where $Z=\sum_{y\in \Omega }exp\left(\left(\sum_{j\in y,k\in N_{j}}w_{jk}y_{j}y_{k}-\theta_{j}\right)/T\right)$. When T=0, μ is concentrated on the $m(\leq 2^{n})$ attractors of the deterministic dynamics, then, its entropy is defined by:

(9) $$E_{\mu }=E_{attractor}=-\sum_{k=1,\ldots ,m}RABS\left(A_{k}\right)logRABS\left(A_{k}\right),$$

where *m* is the number of the final classes of the Markov transition matrix related to the dynamics (8). When T=+∞, μ is scattered uniformly over Ω and E=nlog2. An example of calculation of Relative Attraction Basin Sizes is given in the Table 1. We will estimate E between T=0 and T=+∞ from the attractor entropy $E_{attractor}$ [51] by using the following approximate equality:

(10) $$E\approx log2^{n}-E_{attractor}$$

*E* serves as robustness parameter, being related to the capacity the genetic network has to return to the equilibrium after endogenous or exogenous perturbations [53,59]. We can more generally prove that the robustness of the network, supposed to be sequentially updated, decreases when the variance of the frustration *F* of the network increases, because of the formula: ∂E/∂Log(|w|)=−VarF, where |w| is the absolute value supposed to be the same for all the non zero interaction weights between the genes of the network [51].

**Figure 5:**
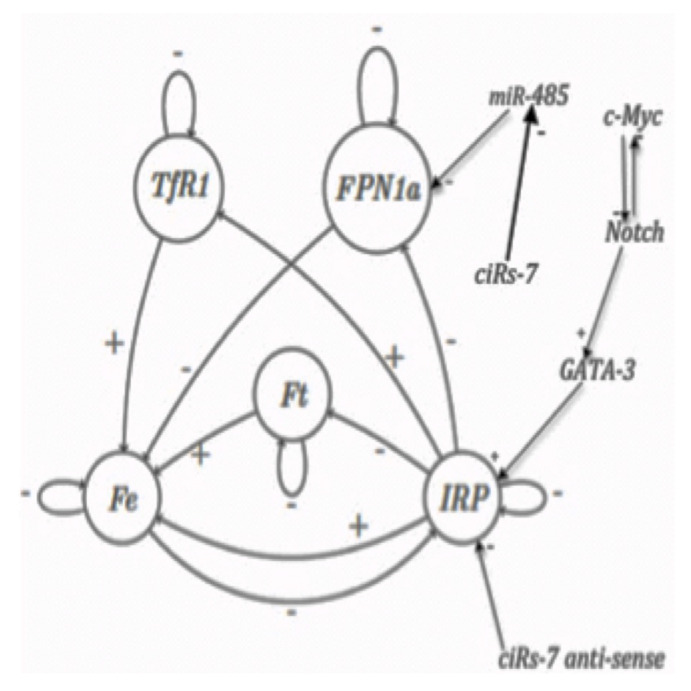
The interaction graph of the iron regulatory network, whose interactions can be activatory (+) or inhibitory (−), such as those of microRNAs, like miR-485.

The regulatory network controlling the iron (Fe) metabolism is given in Figure 5. The central proteins controlling the iron metabolism are iron regulatory proteins IRP1 and IRP2 represented in Figure 5 by a unique entity called IRP, which can be active or inactive, active form only having a regulatory role. In absence of iron, IRP shows a mRNA-binding activity on a specific RNA motif called IRE, and when iron concentration is sufficiently high, it looses its mRNA-binding activity on IRE motif. The iron node (Fe) in Figure 5 represents the cellular iron available for various cellular processes and the node ferritin (Ft) represents a protein capable of internalize thousands of iron ions in a given Ft protein.

The complex mechanism of iron loading into, and release from, ferritin is taken into account here in a very simplified way: ferritin mRNA contains two IRE motifs in its 5’-UTR regions, and IRP consequently inhibits its translation. These IRE motifs are also inhibited by micro-RNAs like miR485, itself inhibited by the cyclic ARN ciRs7. IRP is also directly inhibited by ciRs7-antisense and activated by GATA-3, itself controlled by the positive circuit Notch/c-Myc The transferrin receptor (TfR1) located at the cellular surface allows iron uptake. TfR1 mRNA contains five IRE motifs in its 3’-UTR region, hence, the density of TfR1 receptors at the cell surface is correlated with the IRP activity. Eventually, ferroportin (FPN1a) is an iron exporter located at the cellular surface and contains one IRE motif in its 5’-UTR mRNA region, thus its translation is inhibited by IRP.

By studying the dynamics of the iron regulatory network (supposed to be Boolean, that is with only two states 1 and 0 for each entity, the state 1 corresponding to the proportion of its active form over a certain threshold), it is possible to calculate its asymptotic states (i.e., observed when the time goes to infinity) obtained for any initial condition in attraction basins (Table 1). From this dynamics study, we can then easily calculate $E_{attractor}=2.59412$, hence E=4−2.59412=1.40588, which corresponds to a relatively low robustness, the maximum being equal to four.

The notion of robustness is related to the capacity to diminish the consequences, on the entropy *E*, of endogenous or exogenous variations affecting the common absolute value |w| of the non zero interaction weights of the genetic network. This notion of robustness is closed to that of structural stability in dynamical systems. If the variability of the global frustration *F* of the network is important, then its robustness diminishes. It is for example the case if a gene belonging to a positive circuit [61,62]. of a strong connected component of the interaction graph of the network is randomly inhibited. In this case, the inhibited gene behaves as a sink (i.e., without outing interaction) of state 0, which can enter in contradiction with that predicted by the states and interactions of its neighbors, increasing the variance of *F* and consequently diminishing the dynamic entropy *E*.

### 3.3. Centrality of Nodes and Social Networks

There are four classical types of centrality in a graph (Figure 7). The first is the betweenness centrality [63]. It is be defined for a node *k* in the interaction graph *G* as follows:

(11) $$C_{B}\left(k\right)=\sum_{i\neq j\neq k\in G}\frac{\beta_{ij}\left(k\right)}{\beta_{ij}},$$

where $\beta_{ij}\left(k\right)$ is the total number of shortest paths from node *i* to node *j* that pass through node *k*, and $\beta_{ij}=\sum_{i\neq j\in G}\beta_{ij}\left(k\right)$.

**Figure 6:**
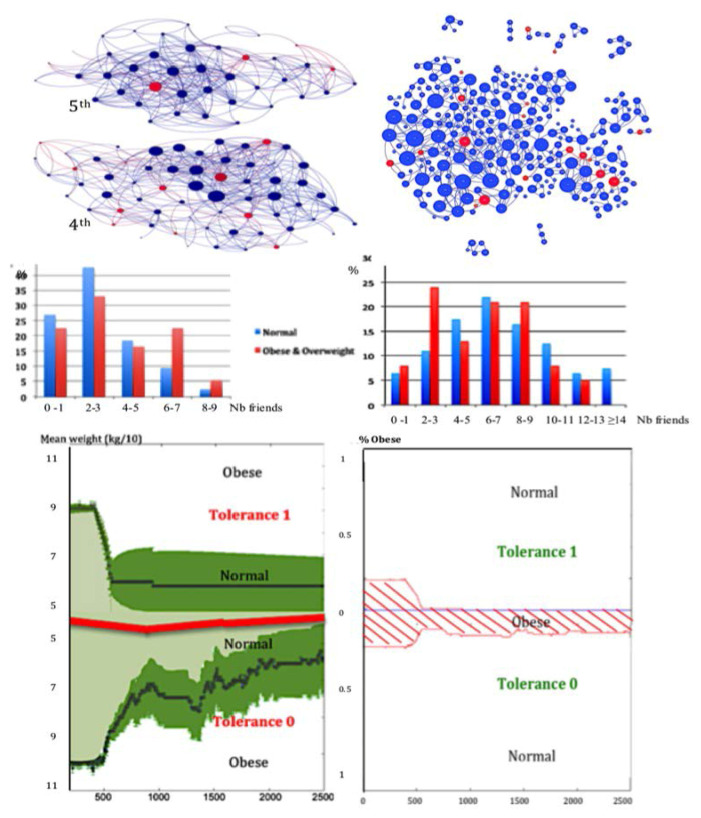
Top left: social graphs related to the friendship relationships between pupils (overweight or obese in red, not obese in blue) of a French high school in 5th and 4th classes, corresponding to ages from 11 to 13 years. Top right: analogue graph for corresponding classes in a Tunisian high school in Tunis. Middle: histograms of the number of friends for pupils from French (left) and Tunisian (right) high schools. Bottom left: mean weight (in black, surrounded by the 95%-confidence interval in green) of pupils coming back to an acceptable “normality”, due to a preventive education of 10% of the betweenness central nodes obese, calculated for two sub-populations of tolerance h = 1 (top) and h = 0 (bottom). Bottom right: percentage of obese in these two sub-populations.

Let consider now a social network with a threshold Boolean dynamics following the Equation (7), containing overweight or obese (state 1) and normal (state 0) individuals as nodes. By concentrating the pedagogic efforts on the betweenness central nodes overweight or obese with tolerance 1, which are “hubs” of the social network, it results a rapid decrease of the number of overweight or obese, more important if the educative effort has been done on individuals with tolerance 1 than with tolerance 0 (Figure 6 Bottom right), as well as a decrease of their mean weight (Figure 6 Bottom left). The second corresponds to the degree centrality, which can be defined from the notions of out-, in- or total degree of a node *i*, corresponding to the number of arrows of the interaction graph, respectively outing, entering, or both, connected to *i*. For example, the in-degree centrality is defined by:

(12) $$C_{i}^{indeg}=\sum_{j=1,\ldots ,n;j\neq I}a_{ij}/\left(n-1\right),$$

where the general coefficient of the adjacency matrix *A* of the graph, denoted $a_{ij}$, equals one if there is a link between *j* and *i*, and else zero.

The third type of centrality is the closeness centrality. The closeness is the reciprocal of the average fairness, which is defined by averaging over all nodes *j* of the network else than *i* a distance between *i* and *j*, equal to $\sum_{j=1,\ldots ,n;j\neq i}d\left(i,j\right)/\left(n-1\right)$, then:

(13) $$C_{i}^{clo}=\left(n-1\right)/\left[\sum_{j=1,\ldots ,n;j\neq i}d\left(i,j\right)\right]$$

where the chosen distance is the length of the closest path between *i* and *j*. The last type of classical centrality is the spectral centrality or eigen-centrality, which takes into account that the neighbors of a node *i* can be also highly connected to the rest of the graph (connections to highly connected nodes contribute more than those of the weakly connected nodes). Hence, the eigen-centrality measures more the global influence of *i* on the network and verifies:

(14) $$C_{i}^{eigen}=\sum_{j=1,\ldots ,n;j\neq i}C_{j}^{eigen}/\lambda$$

where λ is the greatest eigenvalue of the adjacency matrix *A* of the graph.

The four centralities above have each their intrinsic interest: (i) the betweenness centrality of a node is related to its global connectivity with all the nodes of the network, (ii) the degree centralities indicate the level of local connectivity (in-, out- or total) of a node with only its nearest nodes, (iii) the closeness centrality measures the relative proximity a node has with the other nodes of the network for a given distance on the interaction graph of the network, and (iv) the eigen-centrality corresponds to the ability for a node to be connected to possibly a few number of nodes, but having secondarily a high connectivity, which indicates for example an important relay for the dissemination of news in social networks, the social networks being also used to model the spread of rumors, political opinions and social diseases, such as obesity and its most important consequence, type II diabetes.

For example, for curing a social disease, an Hospitaller Order of Saint Anthony has been founded in 1095 at La Motte (presently Saint Antoine near Grenoble in France) by Gaston du Dauphiné, whose son suffered from a fungal disease, known in the Middle Ages as Saint Anthony’s fire or ergotism, caused by a transformation of grain like rye into a drug (the ergotamine) in flour provoking convulsions often leading to death. The members of the Saint Anthony Order were specialized in curing patients suffering from ergotism and they spread with about 370 hospitals over the whole Western Europe in the fourteenth century, able to treat about 4000 patients. They observed that the disease propagation was due to social habits like eating same type of bread, then ergotism is considered as the first social disease whose contagion process depends on alimentation behaviour.

Now obesity is considered as the most characteristic social “contagious” disease, and has been defined as a pandemic (i.e., an epidemic having a world wide prevalence) by the WHO . Both nutrition mimicking and social stigmatization explain the dissemination of obesity into a social network [64,65]. Obesity consists in an excessive accumulation of fat in adipose tissue favoring chronic diabetic and cardiovascular diseases. In UK for example, 36% of men and 33% of women are predicted to be obese in 2030 for only 26% of both sexes in 2010 [66,67,68,69] showing the critical character of the present explosion of obesity, especially in young people. For assessing the prevalence of the disease in young population, we have observed social graphs based on the existence or not of friendship between pupils in the 5th and 4h classes of two high schools (corresponding to ages from 11 to 13 years), one in France at Jœuf already published in [5], and a new one in Tunisia at Tunis. The corresponding graphs (Figure 6 Top) and the histograms of the number F of friends of the pupils, show in both cases that the distribution of F is uni-modal for normal weight pupils and bimodal for overweight or obese ones (Figure 6 Middle), because of a double phenomenon in obese pupils: a part of them attracts with a friendship link due to their open personality, and another part repulses. Then, we have done the simulation of a preventive education of central obese nodes following WHO recommendations [70] during the dynamics of obesity spread following the Equation (7), in which we give the tolerance h = 0 (resp. h = 1) to the obese of the first (resp. second) mode. This education has been given in Figure 6 to the obese central nodes for betweenness in the social graph G. The result is the best between the four centralities defined above, but it remains an irreducible percentage of obese in the population of tolerance zero (Figure 6 Bottom right). For that reason, we need to introduce a new notion of centrality based on the notion of entropy.

## 4. Discussion

### 4.1. The Notion of Entropy Centrality

**Figure 7:**
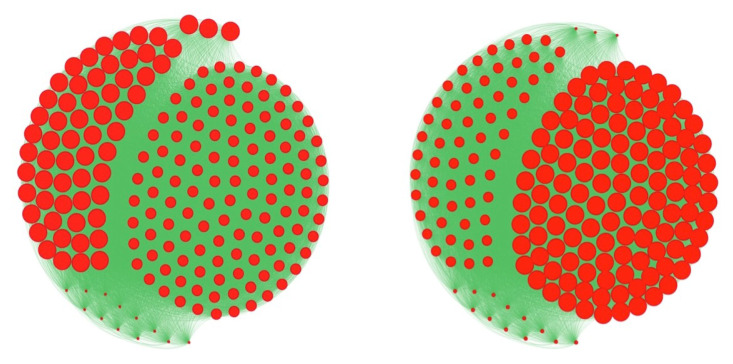
Comparison between two classical types of centrality in the graph of the Tunisian high school between eigenvector (left) and total degree (right) centralities (node size is proportional to its centrality).

**Figure 8:**
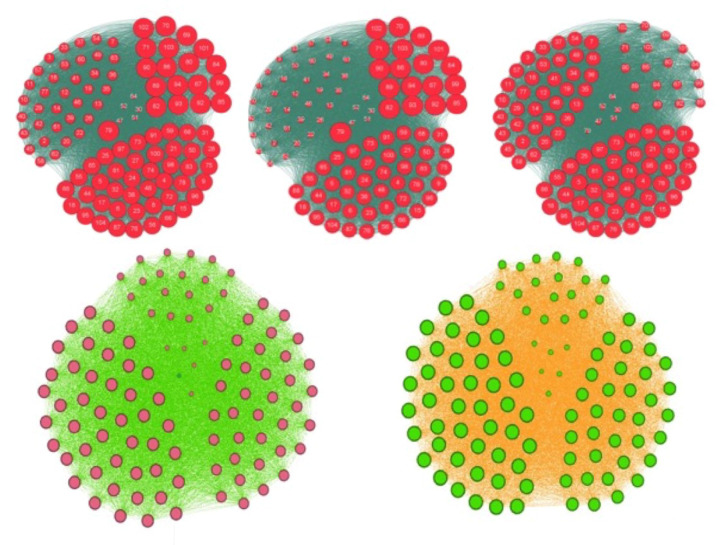
Top: representation of the whole graph of the French high school. The size of the nodes corresponds to their centrality in-degree (left), eigenvector (middle) and total degree (right). Bottom: threshold for a therapeutic education leading back to the normal weight state the N obese individuals having the entropic centrality maximum: after stabilization of the social network dynamics, we get all individuals overweight or obese in red (left) if N = 20 and all individuals normal in green (right) if N = 21, this number constituting the success threshold of the education.

Indeed, despite the interesting properties of the various classical centralities (Figure 6, Figure 7 and Figure 8), the therapeutic education targeting the most central nodes leaves in general a not-neglectible percentage of obese after education, then we will discuss now the introduction of a new notion of centrality, called the entropy centrality, taking into account the heterogeneity of the vector having as components the state and the tolerance, into the neighbor set of the node *i*, and not only the connectivity of the graph:

(15) $$C_{i}^{entropy}=-\sum_{k=1,\ldots ,s}\nu_{k}Log\nu_{k},$$

where $\nu_{k}$ denotes the *k*th frequency among *s* of the histogram of the values of the vector (state, tolerance) observed in the neighborhood $V_{i}$, set of the nodes linked to the node *i*.

This new notion of centrality is more useful than the others to detect the good candidates to be educated in order to transform their state to the normal state 0, because they can influence more efficiently a heterogeneous environment to recover the normality. An illustration of this fact is done in the example of the Section 3.3.

By comparing the results obtained thanks to a preventive and therapeutic education leading the overweight or obese nodes to the normal weight, we observe that with only the 21 individuals the most entropic central educated (Figure 8 Bottom right), all the population of pupils is going to the normal weight state, but we need 68 individuals with the total degree, and 85 individuals with the in-degree and eigenvector centralities (Figure 8 Top and Bottom left). Then, the best public health policy against the obesity pandemics consists in using the notion of entropy centrality to select the targets of the therapeutic education [5].

### 4.2. The Mathematical Problem of Robustness and the Notion of Global Frustration

Each of the examples presented in Section 3 have used the dynamics governed by the deterministic Hopfield transition of the Equation (7) [71]. We have exhibited complex multi-attractor asymptotic behaviors and the central problem to discuss now is the conservation of the attractor organization (namely the number of attractors, the size of their attraction basins, and their nature, i.e., fixed configurations or limit-cycles corresponding to oscillatory states).

This problem of robustness of the attractor landscape is related to the architecture of the interaction graph G(J) linked to the (discrete or continuous) Jacobian matrix *J*. The problem of robustness has been often considered since 50 years [41,72] under different names (bifurcation, structural stability, resilience, etc.) and it is still pertinent [73,74].

If we consider the Hopfield rule with a constant absolute value w>0 for its non-zero interaction weights, we can study the robustness of the network with respect to the variations of w, by using the two following propositions [51,52]:

**Proposition** **1.** *Let us consider a deterministic Hopfield Boolean network which is a circuit sequentially or synchronously updated with constant absolute value w for its non-zero interaction weights. Then, its dynamics is conservative, keeping constant on the trajectories the Hamiltonian function G defined by:*

(16) $$G\left(x\left(t\right)\right)=\sum_{i=1,\ldots ,n}{\left(x_{i}\left(t\right)-x_{i}\left(t-1\right)\right)}^{2}/2=\sum_{i=1,\ldots ,n}{\left(g\left(w_{i(i-1)modn}x_{i-1}\left(t-1\right)\right)-x_{i}\left(t-1\right)\right)}^{2}/2,$$

*where g is the Heaviside function. G(x(t)) is equal to the total discrete kinetic energy, and to the half of the global dynamic frustration $F\left(x\left(t\right)\right)=\sum_{i=1,\ldots ,n}F_{i,(i-1)modn}\left(x\left(t\right)\right)$, where $F_{i,(i-1)modn}$ is the local dynamic frustration related to the interaction between the nodes (i−1) and i: $F_{i,(i-1)}=1$, if sign $\left(w_{i(i-1)}\right)$ = 1 and $x_{i}\left(t\right)\neq x_{i-1}\left(t-1\right)$ or sign $(w_{i(i-1)})=-1$ and $x_{i}\left(t\right)=x_{i-1}\left(t-1\right)$, else $F_{i,(i-1)}=0$.*

**Figure 9:**
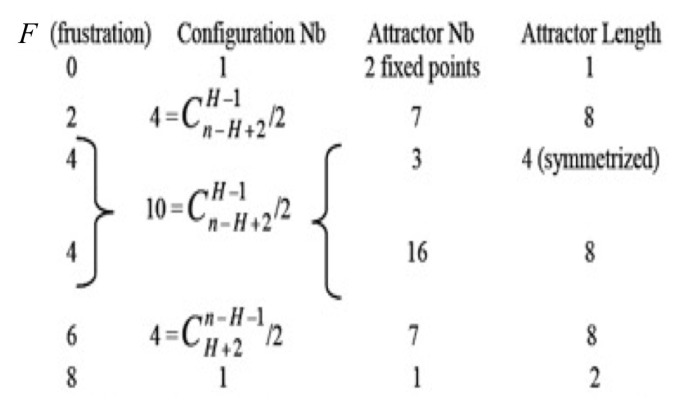
Description of the attractors of circuits of length 8 for which Boolean local transition functions are either identity or negation.

The result of the Proposition 1 holds if the network is a circuit for which transition functions are Boolean identity or negation [75] on which we can easily calculate the global frustration F and show that it characterizes the trajectories and remains constant on them (Figure 9). Then, consider now the dynamic entropy *E* of a Hopfield network [53,76] as a robustness parameter quantifying the capacity the network has to return to its equilibrium measure μ on the configuration space $\Omega ={\left\{0,1\right\}}^{n}$ after a perturbation. We can calculate *E* by using the following equality:

$$E=E_{\mu }-E_{attractor},$$

where $E_{\mu }=-\sum_{x\in \Omega }\mu_{x}log\mu_{x}$, $\mu_{x}=\mu \left(\left\{x\right\}\right)$ being the value of the invariant measure μ at the configuration *x*, and $E_{attractor}=-\sum_{k=1,m\leq 2^{n}}\mu \left(C_{k}\right)log\mu \left(C_{k}\right)$, *m* being the number of the network attractors and $C_{k}$ the attraction basin of the attractor $A_{k}$.

∂E/∂w, which denotes the derivative of the dynamic entropy *E* with respect to the absolute value of the weight *w*, can be considered as a new robustness parameter. We have: $\partial E/\partial w=\partial E_{\mu }/\partial w-\partial E_{attracteur}/\partial w$ and we can prove the following result [8,51]:

**Proposition** **2.** *In the parallel updating mode of a Hopfield network, we have:*

$$\partial E_{\mu }/\partial w=-wVar_{\mu }\left(F\right),$$

*where $Var_{\mu }$ is taken for the invariant Gibbs measure μ defined by: $\mu_{x}=exp\left(\sum_{i\in x,j\in y}w_{ij}x_{i}y_{j}\right)/Z$, with $Z=\sum_{x\in \Omega ,y\in \Omega }exp\left(\sum_{i\in x,j\in y}w_{ij}x_{i}y_{j}\right)$.*

**Figure 10:**
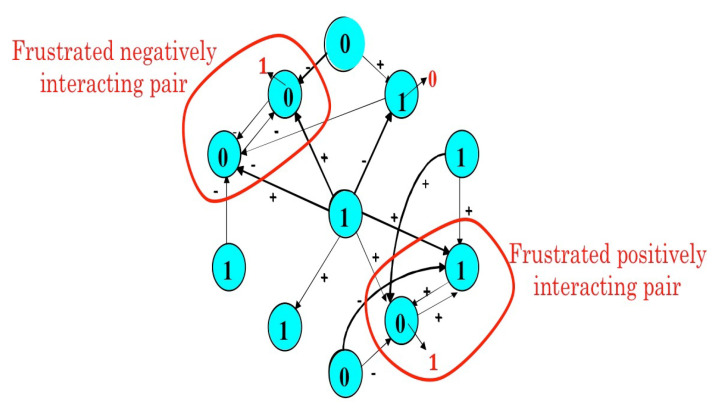
Frustrated pairs of nodes belonging to positive circuits of length 2 in the genetic network controlling the flowering of Arabidopsis thaliana. The network evolves by diminishing the global frustration until the attractor (here a fixed configuration, whose last changes are indicated in red) on which the frustration remains constant.

The Proposition 2 indicates that there is a close relationship between the robustness (or structural stability) with respect to the parameter w and the global dynamic frustration *F*. This global dynamic frustration *F* is in general easy to calculate. For the configuration *x* of the genetic network controlling the flowering of Arabidopsis thaliana [77], there is only two frustrated pairs, hence F(x)=2 (Figure 10).

More generally, all the energy functions introduced in the examples are theoretically calculable and could serve as in physics for quantifying the structural stability. When the number of states in a discrete state space E is too large, or when the integrals on a continuous state space E are difficult to evaluate, then Monte Carlo procedures can provide a good estimation of functions like the dynamic entropy or the variance of the global frustration.

## 5. Conclusions and Perspectives

We have defined some new tools based on the notion of entropy common to both continuous and discrete dynamical systems, based on thermodynamic concepts. These notions are useful in biological modeling to express in which way precise energy functions related to the various concentrations or population sizes involved in the transition equation of a biological dynamical system can be conserved or dissipated along the trajectories until their ultimate asymptotic behavior, the attractors. Future works could be done in the framework of more general random systems [78,79] in which the notions of entropy, invariant measure and stochastic attractors (called also confiners in [54]) are classical to interpret the data observed in all the fields (neural, genetic and social) considered in the present study and use the interpretations they allow in practical applications (see for example [19,80] for some biomedical applications).

## Figures and Tables

**Table 1:** Recapitulation of the attractors of the iron regulatory network of Figure 5, for the parallel updating mode, with the list of expressed (in state 1) and not expressed (in state 0) genes and their relative attraction basin sizes, obtained by simulating in parallel updating mode a threshold Boolean network having a constant absolute value of non zero weights equal to one and a threshold equal to zero, from all the possible initial configurations.

Order	Gene	Fixed Point	Fixed Point 2	Limit-Cycle 1			Limit-Cycle 2	
1	TfR1	0	0	0	0	1	1	0
2	FPN1a	0	0	0	0	0	0	0
3	C-Myc	0	1	0	0	0	0	0
4	Notch	0	0	1	1	1	1	1
5	GATA-3	0	0	1	1	1	1	1
6	IRP	0	0	0	1	0	0	1
7	Ft	0	0	0	0	0	0	0
8	Fe	0	0	0	0	1	0	1
9	MiR-485	0	0	0	0	0	0	0
10	CiRs-7 anti-sense	0	0	0	0	0	0	0
	Relative Attraction Basin Size	512/1024	256/1024	216/1024			40/1024	
